# PCB 118 Exposure Modulates Chromatin Organization, Ribosome Biogenesis, and Autophagy-Related Pathways in Neuron-like: A Transcriptomic Analysis

**DOI:** 10.3390/ijms27115058

**Published:** 2026-06-03

**Authors:** Simone D’Angiolini, Serena Silvestro, Luigi Chiricosta, Michele Scuruchi, Aurelio Minuti

**Affiliations:** 1 IRCCS Centro Neurolesi “Bonino-Pulejo”, Via Provinciale Palermo, Contrada Casazza, 98124 Messina, Italy; 2 Department of Clinical and Experimental Medicine, University of Messina, 98124 Messina, Italy

**Keywords:** environmental pollutants, polychlorinated biphenyls (PCBs), PCB 118, RNA-seq, neurotoxicity mechanism

## Abstract

Polychlorinated biphenyls (PCBs) are persistent environmental pollutants associated with neurodevelopmental and neurodegenerative disorders. PCB 118 is one of the most abundant congeners and exerts neurotoxic effects, yet the molecular mechanisms underlying its impact on human neurons remain poorly understood. We investigated the molecular response of retinoic acid-differentiated, neuron-like SH-SY5Y cells exposed to 5 µM PCB 118 for 24 h, a concentration that did not affect cell viability. RNA sequencing identified 1239 differentially expressed genes. Functional enrichment and protein-protein interaction analyses identified upregulation of histone and chromatin structural genes, indicative of substantial chromatin remodeling. In parallel, a significant downregulation of genes involved in ribosome biogenesis and rRNA processing was observed, potentially indicating impairment of the protein synthesis machinery. These transcriptional changes point to a coordinated reprogramming of nuclear architecture and translational machinery, potentially compromising neuronal homeostasis. The modulation of proteostasis-related pathways further supports a mechanistic link between PCB 118 exposure and neuronal dysfunction. Our results provide a comprehensive transcriptional framework connecting PCB 118 to chromatin-mediated gene regulation and suppression of ribosome biogenesis in human neuron-like cells. This study offers mechanistic insights into how environmental PCB exposure may contribute to neurotoxicity and highlights molecular pathways potentially implicated in the development of neurodegenerative disorders.

## 1. Introduction

Polychlorinated biphenyls (PCBs) are persistent, highly toxic environmental pollutants that interfere with multiple cellular signaling systems [1,2]. They are employed in hydraulic systems, electrical equipment, and as lubricants and coolants and in capacitors and transformers [3]. Human exposure to PCBs occurs mainly through the diet, particularly through the consumption of contaminated fatty foods, fish, meat, and dairy products [4,5]. Toddlers and children may be especially vulnerable to elevated inhalation exposure, mainly due to PCBs in indoor air [6]. For young children, contact with the skin or accidental ingestion of PCB-contaminated soil or dust may also represent a significant exposure risk [3].

As semi-volatile substances, PCBs can be present in both gaseous and particulate forms [7]. Possible major sources of indoor PCB inhalation exposure include older caulking materials, paints, and fluorescent light ballasts manufactured before 1979 [3]. Beyond these legacy sources, PCBs may also originate from non-legacy sources, such as the unintentional contemporary production of PCBs in pigments used in paints and various consumer products [8]. Buildings containing PCB-related materials may also release PCBs into the outdoor air [9]. Other major sources of PCBs in outdoor air include hazardous waste sites, improper or illegal disposal of industrial waste and consumer products, leaks from electrical transformers, waste incineration, and volatilization from water bodies over contaminated sediments [10]. PCBs are introduced into soils mainly through atmospheric deposition [11].

Exposure to PCBs has been linked to the development of several chronic diseases, including endocrine dysfunction, type 2 diabetes, cardiovascular disease, obesity, liver disorders, and neurological deficits [12,13,14,15,16,17]. In addition, these compounds adversely affect the immune, reproductive, nervous, and endocrine systems and are classified as carcinogenic [1]. Despite the ban on PCB use established by the 2001 United Nations Environment Programme Stockholm Convention on persistent organic pollutants (POPs), these compounds remain ubiquitous as POPs and continue to represent a significant threat to global health [2]. This persistence is largely due to their high lipophilicity and resistance to degradation, properties that arise from their inherent chemical stability [18].

Because of their lipophilic nature, PCBs accumulate in adipose tissues and may reach lipid-rich organs, including the central nervous system (CNS) [19,20]. Chronic exposure to these compounds has been linked to impaired neurological development and an increased risk of neurodegenerative diseases [21,22,23]. Based on their structure, PCB congeners are commonly classified into two main categories: non-dioxin-like (NDL-PCBs) and dioxin-like (DL-PCBs) [24]. DL-PCBs are of particular relevance in neurodegeneration due to their neurotoxic effects and disruption of neuronal development and function [25]. PCB 118 is one of a select group of 12 congeners (PCBs 77, 81, 105, 114, 118, 123, 126, 156, 157, 167, 169, and 189) classified as DL-PCBs. These 12 congeners have chemical-physical and toxicological properties comparable to those of dioxins and furans. Due to their coplanar structure, DL-PCBs, including PCB 118, act as ligands for the aryl hydrocarbon receptor (AhR), the canonical receptor for 2,3,7,8-tetrachlorodibenzo-p-dioxin (TCDD), thereby triggering AhR-mediated toxic responses [26]. The developmental neurotoxic effects of PCBs have been strongly supported by findings from experimental animal studies. In contrast, although less extensively investigated, evidence suggests that PCB exposure may also influence the risk, timing, and severity of affective disorders in adults, as well as late-onset neurodegenerative diseases [17].

In experimental models, PCB 118 has been associated with oxidative stress, altered endocrine and neurodevelopmental signaling, mitochondrial dysfunction, and transcriptional dysregulation, supporting its relevance as a model compound for investigating pollutant-induced neuronal stress responses [3,27,28].

The association between PCB exposure and neurodegenerative diseases such as Parkinson’s disease and Alzheimer’s disease (AD) is biologically plausible, as the high lipid content of the human brain facilitates PCB accumulation. Indeed, several studies have shown that PCB exposure is associated with impaired cognitive function, particularly affecting verbal abilities, learning, and short-term memory, in both children and adults [29,30]. Moreover, senile dementia may result from cardiovascular conditions such as hypertension, which has been linked to PCB exposure [31,32].

Our previous study found that PCB 153, a NDL-PCB, can disrupt critical neuronal functions, particularly those governing protein degradation and pathways implicated in neurodegeneration, even without causing overt cytotoxicity [33]. While our previous research focused on PCB 153, it became necessary to extend this investigation to PCB 118 to address a critical knowledge gap. As one of the most abundant dioxin-like congeners in the food chain, PCB 118 provides an ideal model to evaluate how AhR-mediated molecular mechanisms influence chromatin remodeling, an aspect that remains less explored compared to NDL congeners.

**Figure 1:**
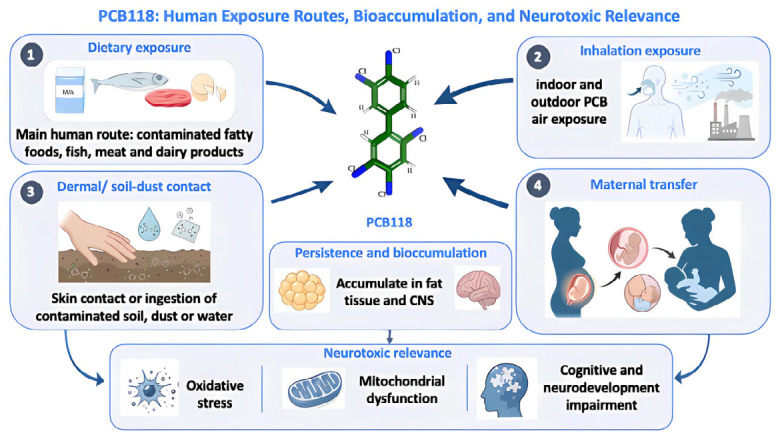
Schematic overview of the main sources of PCB exposure and their potential impact on neuronal cells. PCBs derive from environmental and indoor sources, enter the human body mainly through diet, inhalation, and contact with contaminated dust or soil, accumulate in lipid-rich tissues including the CNS, and may promote oxidative stress, mitochondrial dysfunction, and transcriptional dysregulation in neurons. This image was created using the image bank of Servier Medical Art (available online: http://smart.servier.com/, accessed on 14 May 2026), licensed under a Creative Commons Attribution 4.0 (CC BY 4.0), available online: https://creativecommons.org/licenses/by/4.0/ (accessed on 14 May 2026). Chemical structures depicted in the figure were generated using the PubChem database (accessed on 14 May 2026). PCB, polychlorinated biphenyl; CNS, central nervous system; DL-PCBs, dioxin-like polychlorinated biphenyls; AhR, aryl hydrocarbon receptor.

Although PCB 118 and other dioxin-like PCBs have been associated with neurotoxic and neurodevelopmental effects (Figure 1), the molecular responses induced by PCB 118 in differentiated human neuron-like cells remain incompletely understood. To investigate this, a model of differentiated human SH-SY5Y neurons was employed, and RNA sequencing (RNA-seq) analysis was performed to define the transcriptional response induced by exposure to PCB 118. SH-SY5Y cells represent a human neuroblastoma-derived cell line widely used as an in vitro model for studying neurobiological processes, neurotoxicity, and molecular mechanisms associated with neurodegenerative diseases [34,35]. Their ability to differentiate into a neuronal phenotype makes them particularly useful for investigating the effects of toxic substances on mature neurons. This approach allows for a precise characterization of PCB 118-induced alterations in chromatin remodeling and gene expression mechanisms that remain largely unexplored in traditional, non-differentiated cellular contexts. In light of the growing research focus on PCB neurotoxicity, this study aimed to delineate the biological pathways and gene networks that mediate the neurotoxic effects of PCB 118.

## 2. Results

### 2.1. Cell Viability Assay

**Figure 2:**
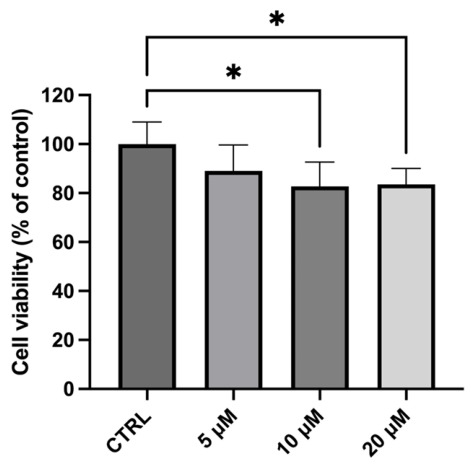
Effect of PCB 118 on the viability of differentiated SH-SY5Y cells. Cells were exposed for 24 h to increasing concentrations of PCB 118 (5, 10, and 20 µM), and viability was subsequently measured. Values represent the mean ± standard deviation from eight independent experiments; * *p* < 0.05 versus untreated control (CTRL).

Cell viability (3-(4,5-dimethylthiazol-2-yl)-2,5-diphenyltetrazolium bromide assay, MTT) was approximately 89% at 5 µM, 82.8% at 10 µM, and 83% at 20 µM PCB 118 compared with untreated control cells (CTRL). Although viability remained above 80% at the higher concentrations, 5 µM was the only concentration that did not significantly reduce cell viability. Therefore, this dose was selected to investigate early molecular responses occurring before overt cytotoxicity (Figure 2).

### 2.2. Transcriptomic Data

**Figure 3:**
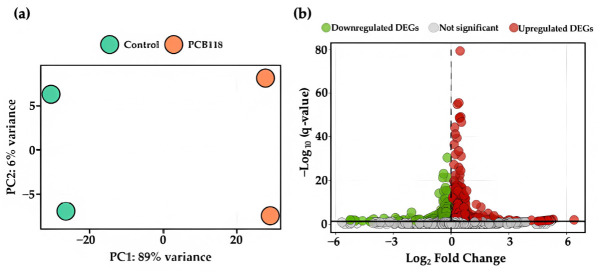
Comprehensive transcriptomic profiling following PCB 118 exposure. (**a**) Principal Component Analysis (PCA) based on normalized read counts, demonstrating the clustering of biological replicates (*n* = 2 per group). The first principal component (PC1), explaining 89% of the total variance, clearly discriminates Control (light green) from PCB 118-treated (orange) SH-SY5Y cells. (**b**) Volcano plot representing the differential expression analysis of all identified transcripts. The x-axis denotes the Log_2_ Fold Change (FC), and the y-axis reflects the statistical significance (−Log_10_ q-value). The horizontal solid line indicates the significance threshold (q-value < 0.05) while the vertical dashed line separates downregulated DEGs from upregulated ones.

To investigate the global transcriptomic changes induced by PCB 118 exposure, we first performed a Principal Component Analysis (PCA) on the normalized expression data. This multivariate approach allowed us to visualize the clustering patterns and to identify the primary drivers of experimental divergence. As shown in Figure 3a, a clear segregation between the CTRL and treated groups was observed along the first principal axis (PC1), which explained 89% of the total variance. Such a distribution confirms that the treatment was the predominant source of molecular variance, with biological replicates demonstrating high intra-group reproducibility. Following the initial assessment, differential expression analysis was performed on RA-differentiated SH-SY5Y cells subjected to 5 µM PCB 118 for 24 h. DEGs were identified based on a significance threshold of q-value < 0.05. Notably, no fixed fold change (FC) filter was imposed to maintain a broad and unbiased overview of the biologically relevant fluctuations. This analysis revealed a total of 1239 DEGs, including 675 upregulated and 564 downregulated transcripts. Detailed statistics for all identified genes are available in Supplementary Table S1. The overall distribution of the magnitude and statistical significance (−log_10_ q-value) of these expression changes is depicted in the Volcano Plot (Figure 3b).

To characterize the functional landscape of the identified DEGs, an Over-Representation Analysis (ORA) was performed independently on the global DEGs dataset and also using just the upregulated and downregulated DEGs subsets. Statistical significance was determined using the Benjamini-Hochberg method to control the FDR, with a significance threshold of q-value < 0.05. Initial analysis of the total DEGs pool identified 6 significantly enriched Gene Ontologies (GO) terms among the different types of GO: biological process (BP), molecular function (MF) and cellular component (CC). Functional stratification of the DEGs revealed a distinct asymmetry in the transcriptional response. Analysis of the downregulated subset indicated a highly localized functional impact, with only 3 BP reaching statistical significance (Table 1).

In contrast, the upregulated gene set exhibited a significantly broader reach, yielding 60 enriched BPs. Within this expansive set, 6 global ontologies were found to be entirely nested; these core terms were prioritized and reported at Table 2.

**Figure 4:**
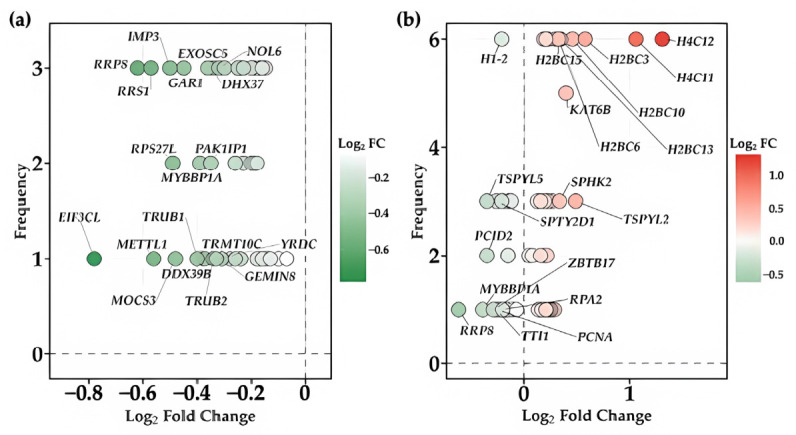
Functional mapping of unique DEGs following Gene Ontologies (GO) over-represented. (**a**) Dot plot showing 56 unique DEGs associated with downregulated GO terms. (**b**) Dot plot showing 63 unique DEGs associated with the global and upregulated GO Over-Representation Analysis (ORA). For both plots, the x-axis displays log_2_ fold change values, and the y-axis reflects the number of enriched biological processes to which each DEG is assigned. Symbol colors distinguish between downregulated (green) and upregulated (red) DEGs.

The functional enrichment analysis reveals a coordinated modulation of cellular machinery, specifically targeting pathways involved in molecular biogenesis and the structural organization of chromatin. As summarized in Table 1, the enriched ontologies include Ribonucleoprotein complex biogenesis (39 genes), Ribosome biogenesis (28 genes), and ncRNA processing (37 genes). The robust representation of these categories indicates a synchronized downregulation of the protein synthesis apparatus, suggesting that the cellular response involves a coordinate reduction in translational machinery and ncRNA maturation processes. By merging the DEGs involved in the ontologies mentioned above, we obtained a unique list of 56 DEGs; their expression patterns are briefly illustrated in Figure 4a, while all the information related to this is fully provided in the Supplementary Table S2. Parallel to this process, the analysis identified a cluster of six positive ontologies that resulted from both ORA analysis based on all the DEGs and just from the upregulated DEGs. To achieve higher precision and a more comprehensive overview, we integrated the downregulated genes identified in the global enrichment analysis into this structural framework. The key processes identified in this context and reported in Table 2 reflect a targeted impact on the spatial and temporal arrangement of the nuclear architecture. Specifically, the enrichment in nucleosome-related terms and chromatin remodeling suggests a dynamic shifting in DNA packaging, which serves as a primary epigenetic gatekeeper for transcriptional access. The inclusion of structural constituents and protein–DNA complexes further confirms a targeted modulation of histone proteins and other scaffolding components that maintain genomic integrity. Merging all the DEGs involved in the 6 ontologies above, we obtained a list of 63 unique DEGs. Integrating the DEGs from the aforementioned ontologies yielded a unique set of 63 genes; while their expression profiles are highlighted in Figure 4b, a comprehensive dataset containing all associated values is provided in Supplementary Table S3.

**Figure 5:**
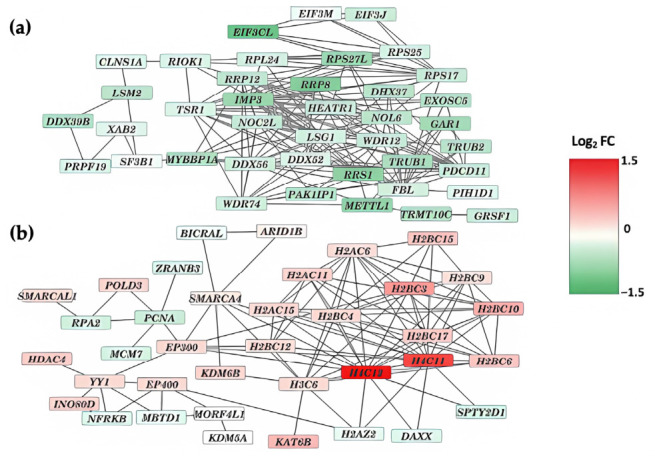
Protein-Protein Interaction (PPI) networks and functional connectivity of DEGs. (**a**) Interaction network derived from the downregulated GO term enrichment; of the 56 initial DEGs, 40 functional nodes are represented based on known biological interactions. (**b**) Global interaction network encompassing the overall DEG list, featuring 37 interconnected nodes derived from the 63 unique DEGs identified. Node color indicates the log_2_ fold change, with red representing upregulation and green representing downregulation.

To bridge the functional enrichment results from the unique lists of DEGs to a structural understanding of protein interactions, both the lists obtained were analyzed using the STRING database. To ensure the highest level of biological robustness and to eliminate “noise” or false-positive associations often present in high-throughput datasets, we applied a maximum stringency threshold. Specifically, a minimum required interaction score of 0.900 (maximum confidence) was mandated, alongside a high-confidence FDR of 0.01. Furthermore, the interaction sources were selectively filtered to include only “Experiments” and “Databases”. This choice enhances the reliability of the analysis by prioritizing physically or biochemically validated interactions, while excluding associations derived from text mining, co-expression, or computational predictions. As a result, the generated networks more accurately reflect established biological relationships rather than predicted interactions (Figure 5).

The identification of these two distinct interaction networks provides a structural framework for the biological processes previously described. While the downregulated and overall sets differ in their internal connectivity, both point toward a focused cellular response to PCB 118. These findings will be further discussed in the context of SH-SY5Y homeostasis, evaluating how PPI interaction hubs may contribute to the observed phenotype.

To further explore the biological impact of PCB 118 exposure, we performed a Gene Set Enrichment Analysis (GSEA) focusing on Hallmark and Reactome datasets. Despite applying a standard significance threshold (q-value < 0.05), no pathways reached statistical significance in these specific databases. This lack of enrichment in Hallmark and Reactome might be attributed to the highly specific nature of the transcriptional changes induced by PCB 118 in our neuron-like model, which may not align with the broader or more curated functional categories defined in these sets.

**Figure 6:**
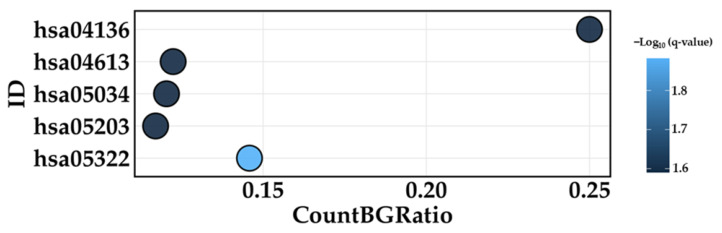
The dot plot illustrates the KEGG pathway enrichment analysis of differentially expressed genes (DEGs) in SH-SY5Y cells following PCB 118 exposure. The y-axis lists the enriched biological pathways, while the x-axis represents the CountBGRatio, indicating the ratio of DEGs associated with a specific pathway relative to the total number of genes in that pathway’s background. The color gradient represents the statistical significance, calculated as −log10(q-value).

Conversely, an enrichment analysis conducted using the KEGG database revealed several significantly modulated pathways, as shown in Figure 6.

Detailed data for all the pathways represented in Figure 6 are available in Supplementary Table S4. Specifically, the pathway exhibiting the highest background ratio is ‘Au-tophagy—other’ (hsa04136). In our model, this pathway involves 8 significantly modulated genes. Among these, *ATG12*, *ATG5*, *ATG7*, *BECN1*, *PIK3R4*, and *PPP2CB* were found to be downregulated. Furthermore, *IGBP1* and *RPTOR* were also significantly upregulated, suggesting a comprehensive inhibition of the autophagic machinery following PCB 118 exposure. A graphical summary of the changes to the pathway is provided in Supplementary Figure S1.

### 2.3. Western Blot Analysis of Selected Autophagy-Associated and Oxidative-Stress-Related Markers

**Figure 7:**
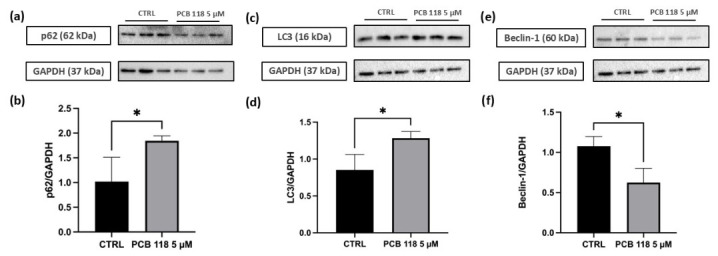
PCB 118 exposure alters the expression of key autophagy-related proteins. Representative Western blots and corresponding densitometric analysis of p62 (**a**,**b**), total LC3 (**c**,**d**), and Beclin-1 (**e**,**f**) in control (CTRL) cells and cells exposed to PCB 118 (5 μM). Protein levels were normalized to GAPDH. PCB 118 treatment significantly increased p62 and total LC3 expression, while significantly reducing Beclin-1 levels compared with CTRL, supporting changes in selected autophagy-associated markers. Data are presented as mean ± SD. * *p* < 0.05 vs. CTRL.

To explore whether PCB 118 exposure was accompanied by changes in selected autophagy-associated markers, we examined p62, LC3, and Beclin-1 protein levels by Western blot analysis. These proteins were selected as representative markers of autophagy-related responses; however, their expression alone does not allow a definitive assessment of autophagic flux. PCB 118 exposure induced a significant increase in p62 levels compared with the control group (Figure 7a,b). Since p62 is normally degraded during autophagic processing, its accumulation may indicate impaired autophagic turnover.

In parallel, total LC3 immunoreactivity was significantly increased in PCB 118-treated cells relative to controls (Figure 7c,d). By contrast, Beclin-1 levels were significantly reduced following PCB 118 treatment (Figure 7e,f), suggesting an impairment of the upstream regulatory events involved in autophagy initiation.

Taken together, these findings reveal that PCB 118 induces a marked dysregulation of autophagy-related pathways, characterized by the accumulation of p62 and LC3 alongside the downregulation of Beclin-1. Overall, these findings indicate that PCB 118 exposure is associated with changes in selected autophagy-related markers. However, because autophagic flux was not dynamically assessed using lysosomal inhibitors or tandem fluorescent LC3-based approaches, these data cannot distinguish between increased autophagosome formation and impaired autophagosome clearance. Full, uncropped membranes for each analyzed protein are shown in Supplementary Figures S2–S4.

**Figure 8:**
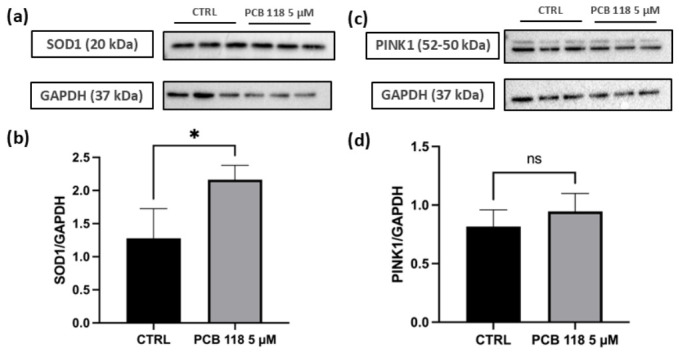
PCB 118 selectively modulates SOD1 expression without significantly affecting PINK1 levels. Representative Western blots and corresponding densitometric analysis of SOD1 (**a**,**b**) and PINK1 (**c**,**d**) in control (CTRL) cells and cells exposed to PCB 118 (5 μM). Protein levels were normalized to GAPDH. PCB 118 treatment significantly increased SOD1 expression, whereas PINK1 levels were not significantly different from those observed in CTRL cells. Data are presented as mean ± SD/SEM. * *p* < 0.05 vs. CTRL; ns, not significant.

To further characterize the molecular response induced by PCB 118 (5 μM), we evaluated the protein expression of SOD1 and PINK1 by Western blot analysis. As shown in Figure 8, PCB 118 exposure resulted in a significant increase in SOD1 protein levels compared with the CTRL group (Figure 8a,b), suggesting the activation of an antioxidant response under treatment conditions.

By contrast, PINK1 expression was not significantly altered following PCB 118 exposure (Figure 8c,d), despite a slight upward trend in the treated group. These findings indicate that, under the present experimental conditions, PCB 118 induces a measurable modulation of the cellular oxidative stress response, as reflected by SOD1 upregulation, whereas PINK1-dependent mitochondrial quality control does not appear to be significantly engaged under these conditions. Full, uncropped membranes for each analyzed protein are shown in Supplementary Figures S5 and S6.

## 3. Discussion

PCBs are recognized as neurotoxic and capable of altering neuronal development and degeneration, affecting signals such as dopamine, inducing oxidative stress, and causing transcriptional changes in various cellular systems [17,36,37]. PCB 118 is a dioxin-like congener associated with cognitive deficits and behavioral alteration, and has been shown to affect gene regulation and neuroendocrine function [38].

In this context, the aim of this study was to investigate the transcriptional and neurotoxic effects of PCB 118 on human SHSY5Y neuronal cells under non-cytotoxic conditions. Indeed, the MTT assay confirmed that 5 µM PCB 118 did not significantly impair cell viability after 24 h exposure, ensuring that the observed transcriptional changes reflect specific molecular responses rather than secondary effects of overt toxicity.

SH-SY5Y cells represent a well-established neuronal model to investigate cellular responses to xenobiotics, including cytoskeletal remodeling and stress-associated signaling pathways [39].

PCA revealed a clear segregation between CTRL and treated samples, with PC1 explaining 89% of the total variance. This strong separation indicates that PCB 118 exposure is the predominant driver of transcriptomic variability. The high intra-group consistency further supports the robustness of the dataset and the reproducibility of the biological effect.

Differential expression analysis identified 1239 significantly modulated genes (q-value < 0.05), of which 675 were upregulated and 564 were downregulated. GO enrichment analysis revealed a strong over-representation of chromatin-related categories among DEGs. Specifically, nucleosome organization, nucleosome assembly, chromatin remodeling, nucleosome, protein–DNA complex, and structural constituent of chromatin were significantly enriched. Nucleosome organization and chromatin remodeling are fundamental processes governing transcriptional accessibility [40], and their modulation implies that PCB 118 may influence gene expression at the level of chromatin structure. This is further supported by the network analysis, where several highly central genes belong to histone families and chromatin structural components. These hub genes likely represent key regulatory nodes coordinating broader transcriptional reprogramming.

Chromatin organization plays a central role in regulating transcriptional accessibility through histone–DNA interactions and dynamic remodeling of nucleosomal structure [41].

In physiologically normal neurons, epigenetic regulatory mechanisms tightly control gene expression programs required for synaptic function, maintenance of cellular homeostasis, and appropriate responses to environmental cues. Chromatin organization and histone-mediated regulation play fundamental roles throughout both the developing and mature CNS. These processes are critically involved in multiple forms of neural plasticity, ultimately contributing to the formation and modulation of complex behavioral traits [42].

Epigenetic changes play a central role in neurodegenerative diseases by disrupting gene regulation without altering DNA, impairing neuronal adaptability and cellular homeostasis [43]. Alterations in these mechanisms can cause genes vital for neuronal activity, causing them to be either inappropriately activated or silenced [44].

In our study, the coordinated upregulation of multiple histone genes, including several *H2B* variants (*H2BC12*, *H2BC13*, *H2BC3*, *H2BC17*, *H2BC10*, *H2BC15*) and H4 variants (*H4C11* and *H4C12*), strongly supports the occurrence of nucleosome remodeling, indicating a dynamic reorganization of chromatin architecture in response to PCB 118 exposure. The canonical H2B histone functions as a key site for multiple DNA repair proteins [45]. Under normal conditions, the protein COMMD4 (Copper Metabolism MURR1 Domain Containing 4) binds directly to unmodified H2B, protecting it from uncontrolled post-transcriptional modifications, particularly monoubiquitylation. However, following the occurrence of a DNA double-strand break, ATM phosphorylates H2B at serine 14, triggering the dissociation of COMMD4 [46,47]. This release permits the RNF20-RNF40 complex to ubiquitylate H2B at lysine 120 within approximately one hour of damage. The resulting H2BK120 ubiquitylation then serves as a platform to recruit critical homologous recombination repair factors, including BRCA1, CtIP, and NBS1, facilitating proper DNA end resection [48,49].

Importantly, we found that the chromatin-associated factor *SET* and the histone acetyltransferase *KAT6B* were upregulated. The *SET* gene encodes a protein that inhibits acetylation of nucleosomes. This protein is primarily located in the endoplasmic reticulum. However, it is also present in the nucleus, where it functions to suppress apoptosis after cells are targeted by cytotoxic lymphocytes. Isoform 1 and isoform 2 strongly inhibit phosphatase 2A activity. They also suppress histone acetylation mediated by EP300/CREBBP and PCAF histone acetyltransferases, likely by blocking access of histone lysine residues to these enzymes. Histone H4 appears to be the main target of this inhibitory effect [50].

KAT6B functions as a histone acetyltransferase and is a central component of the MOZ/MORF multiprotein complex. While its overexpression has been linked to behavioral alterations in animal models [51], its primary molecular role involves the modulation of histone acetylation, specifically H3K9 and H3K23. This activity is likely influenced by its interaction with other components of the KAT6 protein complex, further supporting its role as a key epigenetic regulator [51].

Furthermore, we identified the upregulation of *SPHK2*, which produces one of the two isoforms of sphingosine kinase, enzymes responsible for converting sphingosine into sphingosine 1-phosphate (S1P). S1P is a signaling molecule involved in regulating key cellular functions such as cell movement, growth, and programmed cell death [52].

Consistent with the enrichment of downregulated GO categories related to ncRNA processing, ribonucleoprotein complex biogenesis, and ribosome biogenesis, several key genes involved in ribosomal assembly and maturation were significantly downregulated following PCB 118 exposure. These include *RRP8*, *RRS1*, *GAR1*, *IMP3*, *RPS27L*, *PAK1IP1*, *NOC2L*, *ERAL1*, *LSG1*, *RPL24*, and *URB2*. They constitute an essential group of transacting factors and structural elements required for eukaryotic ribosome biogenesis [53,54,55].

Interestingly, the coordinated downregulation of ribosome biogenesis and RNA processing genes observed in this study is consistent with findings previously reported by our group in an in silico transcriptomic study of mild cognitive impairment and AD [56]. In that study, early and progressive suppression of genes involved in ribonucleoprotein complex biogenesis, rRNA processing, and translation was identified as a shared molecular feature across MCI and AD samples. Notably, several genes that we found downregulated following PCB 118 exposure, including *RPS27L*, *PAK1IP1*, and genes involved in ribosomal subunit maturation and export, such as *RRP8*, *RRS1*, *GAR1*, *IMP3*, *NOC2L*, *ERAL1*, *LSG1*, *RPL24*, and *URB2*, functionally converge on the same biological pathways highlighted in the previous study [56]. The recurrence of translational suppression as a common molecular denominator in both PCB-exposed neuronal cells and early-stage neurodegenerative conditions suggests that impaired ribosome biogenesis may represent a shared vulnerability pathway. In neurons, which rely on tight translational control for synaptic plasticity, even subtle ribosomal perturbations can compromise long-term function. This impairment in protein synthesis directly challenges the cellular proteostasis network—an interconnected system of molecular chaperones, the ubiquitin-proteasome system (UPS), and the autophagy-lysosome pathway (ALP) dedicated to maintaining protein quality [57,58].

In our model, the disruption of ribosomal genes may trigger a compensatory but insufficient response from these proteostatic branches. Specifically, molecular chaperones are essential for a variety of cellular functions, such as assisting protein folding, preventing and reversing aggregation, promoting protein degradation, guiding protein trafficking, and supporting intracellular signal transduction [59]. These chaperones are organized into distinct functional families, such as small heat-shock proteins (sHSPs), *HSP90*, *HSP70*, *HSP60*, and *HSP40*. The transcriptomic shifts observed in our study suggest that PCB 118 might not only suppress the production of new proteins but also strain the existing machinery responsible for protein folding and degradation, potentially leading to proteotoxic stress, a hallmark of neurodegenerative diseases. Under these conditions, cells rely on additional proteostatic mechanisms to cope with the accumulation of misfolded or damaged proteins.

Autophagy is a fundamental and evolutionarily conserved mechanism that preserves cellular homeostasis by breaking down and recycling cellular components, including damaged or misfolded proteins and intracellular pathogens. Autophagy is controlled through a complex sequence of events involving several autophagy-related (ATG) proteins, along with multiple activating and regulatory factors [60]. Autophagy is a vital process that helps maintain proteostasis by breaking down proteins alongside the UPS. While the UPS mainly targets and eliminates individual proteins through the proteasome, autophagy handles the degradation of larger protein aggregates within the lysosome [60]. Among these mechanisms, autophagy represents a critical complementary pathway to molecular chaperones and the UPS.

Defects in autophagy can exacerbate neurodegenerative pathology by allowing the persistence of protein aggregates, defective organelles, and abnormal signaling pathways, contributing to progressive neuronal loss [61]. The initiation of autophagy is regulated by mTOR (mammalian target of rapamycin) and mediated by the Unc-51-like kinase (ULK) complex, which consists of *ULK1*, *FIP200*, *ATG13*, and *ATG101*. This complex is essential for triggering a phosphorylation cascade that activates downstream effectors involved in autophagy. In nutrient-rich environments, mTOR phosphorylates *ULK1* and *ATG13*, thereby inhibiting *ULK1* activity and suppressing the autophagic process [62]. In addition, *ATG13* further suppresses *ULK1* activity, thereby contributing to the inhibition of autophagy initiation [63]. Once autophagy is initiated, *ULK* engages the *PI3KC3* nucleation complex through *ATG101*. This class III phosphatidylinositol 3-kinase complex includes Beclin-1, VPS34, VPS15, ATG14, and AMBRA1, a regulatory activator of BECN1-dependent autophagy [64]. Autophagosome membrane elongation is controlled by two ubiquitin-like conjugation systems that promote LC3 lipidation. In one of these pathways, ATG12 is covalently linked to ATG5 through the sequential activity of the E1-like enzyme ATG7 and the E2-like enzyme ATG10 [65]. Interestingly, we found downregulated *ATG5, ATG12*, and *ATG12* in our transcriptomic analysis as reported in Supplementary Figure S1. This finding may suggest a reduced capacity for autophagosome membrane elongation and LC3 lipidation, potentially indicating an impairment of the autophagy machinery at the transcriptional level. LC3-I is then conjugated to phosphatidylethanolamine, producing LC3-II, which becomes associated with the membrane of the developing phagophore [63].

At the protein level, our findings provide supportive evidence that PCB 118 exposure is accompanied by changes in selected cellular stress markers under non-cytotoxic conditions. In differentiated SH-SY5Y cells, exposure to 5 μM PCB 118 was associated with reduced Beclin-1 expression, together with increased levels of p62 and LC3. This pattern suggests that PCB 118 may affect components of the autophagy-related response; however, it does not allow definitive conclusions regarding autophagic flux. Additionally, our findings are in agreement with previous studies reporting PCB-mediated suppression of autophagy-related pathways [66,67,68,69]. In parallel, increased protein levels of SOD1 suggest activation of an antioxidant response, whereas unchanged PINK1 levels indicate that PINK1-dependent mitochondrial quality control was not robustly engaged under the present experimental conditions. Overall, these protein-level findings are consistent with the presence of a PCB 118-associated cellular stress phenotype, but further targeted and dynamic assays will be required to define the underlying mechanisms [70,71].

Given the potential limitations of this study, our findings should be further confirmed through in vivo models. For instance, the impact of PCBs in vivo may be tissue-specific, and the concentration-dependent effect could differ between in vitro and in vivo models.

Similarly, additional protein markers and chromatin-associated targets would be required to more precisely define the biological consequences of PCB 118 exposure; therefore, future studies are needed.

Although the RNA-seq analysis revealed a clear separation between CTRL and PCB 118-treated samples and identified coherent pathway-level signatures, the transcriptomic findings should be interpreted as hypothesis-generating and require validation in larger independent datasets and complementary experimental models.

Taken together, our findings indicate that exposure to 5 µM PCB 118 induces a broad transcriptional reprogramming in SH-SY5Y neuronal cells even under non-cytotoxic conditions. Transcriptomic profiling revealed that PCB 118 strongly reshapes pathways related to chromatin organization, nucleosomal assembly, and epigenetic regulation, while concurrently suppressing gene networks involved in ncRNA processing, ribosome biogenesis, and RNA maturation. These alterations are supported at the protein level by changes in Beclin-1, p62, LC3, and SOD1, indicating a disruption of autophagy-related pathways and activation of oxidative stress responses. Overall, these data support a mechanistic framework in which PCB 118 promotes an early neuronal stress phenotype characterized by extensive transcriptomic reprogramming, disruption of proteostasis, and activation of oxidative stress responses. These alterations may represent early molecular events that increase neuronal vulnerability to the neurodegenerative process.

## 4. Materials and Methods

### 4.1. Chemical Reagents

PCB 118 used in this study was purchased from LGC Standards (#31508-00-6, LGC Standards, Milan, Italy). Dimethyl sulfoxide (DMSO), used as a solvent for PCB stock solutions and for treating differentiated SH-SY5Y cells, was obtained from Sigma-Aldrich (#D8418, Sigma-Aldrich, Saint Louis, MO, USA). PCB 118 was dissolved in DMSO to prepare a concentrated stock solution (25 mM), aliquoted to avoid repeated freeze–thaw cycles, and stored according to the manufacturer’s recommendations. Immediately before treatment, stock aliquots were diluted in culture medium to obtain the final working concentrations. The final DMSO concentration was kept constant across all experimental conditions, including vehicle controls, and did not exceed 0.1%. All chemicals were sterile filtered prior to use to prevent bacterial contamination.

### 4.2. SH-SY5Y Differentiation and PCB 118 Treatment

The human neuroblastoma cell line SH-SY5Y was sourced from the American Type Culture Collection (ATCC, Manassas, VA, USA). For cell culture, 40,000 cells were distributed into each well of 96-well plates (#353072, Corning Incorporated, Corning, NY, USA), whereas 500,000 cells per well were plated in 6-well plates (#130184, Thermo Scientific, Rochester, NY, USA), using a growth medium based on DMEM/F-12 Ham (#D6421, Sigma-Aldrich, Saint Louis, MO, USA) supplemented with 10% fetal bovine serum (#F7524, Sigma-Aldrich, Saint Louis, MO, USA), 1% penicillin/streptomycin (#P0781, Sigma-Aldrich, Saint Louis, MO, USA), and 1% L-glutamine (#G7513, Sigma-Aldrich, Saint Louis, MO, USA). Cultures were maintained at 37 °C in a humidified incubator with 5% CO_2_. On the day after seeding, neuronal differentiation was initiated by adding 10 µM retinoic acid (RA) (#R2625, Sigma-Aldrich, Saint Louis, MO, USA) and continuing the treatment for 5 consecutive days. Once differentiation was achieved, the cells were challenged for 24 h with a range of PCB 118 concentrations to assess the effects of this congener under controlled experimental conditions.

### 4.3. MTT-Based Cell Viability Assay

Differentiated SH-SY5Y cells were maintained and exposed to PCB 118 in 96-well plates, as previously described in the above section. At the end of the exposure, cell viability was determined by means of the MTT assay using Thiazolyl Blue Tetrazolium Bromide (#M5655, Sigma-Aldrich, Saint Louis, MO, USA). In brief, cultures were incubated for 4 h at 37 °C in maintenance medium supplemented with 0.5 mg/mL MTT, allowing viable cells to convert the tetrazolium salt into insoluble formazan crystals. These crystals were then solubilized for 1 h at 37 °C in isopropanol acidified with 0.1 N HCl, and the optical density was recorded at 570 nm with background subtraction at 630 nm using a BioTek Synergy H1 microplate reader (Agilent, Santa Clara, CA, USA).

### 4.4. Total RNA Processing and cDNA Library Generation

SH-SY5Y cells were grown and exposed to treatments in 6-well plates under the same conditions. At the end of the exposure, cells were harvested by incubation with a 0.25% trypsin–EDTA solution (#T4049, Sigma-Aldrich, Milan, Italy) and collected by centrifugation at 300× *g* for 5 min to obtain compact cell pellets.

Total RNA was isolated from these pellets using the Maxwell^®^ RSC simplyRNA Cells Kit (#AS1390, Promega, Madison, WI, USA) on the Maxwell^®^ RSC automated platform, strictly following the manufacturer’s recommendations. For RNA-seq analysis, two independent biological replicates were prepared for each experimental condition, namely vehicle-treated control cells and cells exposed to 5 µM PCB 118 for 24 h. Each biological replicate was obtained from an independent culture well and was processed separately from cell harvesting through RNA extraction, library preparation, and sequencing.

For transcriptomic profiling, 100 ng of total RNA from each sample were used to generate libraries with the TruSeq^®^ RNA Exome Kit (#20020189, #20020492, #20020183, #20020490; Illumina, San Diego, CA, USA). The resulting libraries were checked for size distribution and integrity on a TapeStation 4150 system (Agilent, Santa Clara, CA, USA) using D1000 ScreenTape reagents (#5067-5582 and #5067-5583). Before sequencing, libraries were denatured with 0.2 N NaOH and diluted to a working concentration of 1.42 pM, then run on an Illumina NextSeq^™^ 550Dx sequencer (Illumina, San Diego, CA, USA) using the NextSeq 500/550 Mid Output Reagent Kit v2.5 (150 cycles) in paired-end configuration.

### 4.5. RNA-Seq–Based Transcriptomic Analysis

To ensure the integrity of the sequencing dataset, raw reads underwent an initial quality assessment using FastQC v.0.12.0 (Babraham Institute, Cambridge, UK) (FastQC. A Quality Control Tool for High-Throughput Sequence Data. Available online: https://qubeshub.org/resources/fastqc, accessed on 1 April 2026). Technical artifacts, including adapter sequences and low-confidence bases, were filtered out through Trimmomatic v.0.40-rc1 [72] to yield a high-quality read set. These processed sequences were mapped to the GENCODE hg38 (v39) reference genome employing the STAR aligner (v.2.7.10a_alpha_220207) [73]. Gene-level quantification was subsequently performed using HTSeq v.0.13.5 [74] to generate raw count matrices. Statistical evaluation of differentially expressed genes (DEGs) was conducted within the R environment (v.4.2.0) using the DESeq2 package (v.1.36.0) [75]. This framework utilizes generalized linear models (GLM) based on a negative binomial distribution to account for the overdispersion inherent in RNA-seq data. *p*-values were adjusted using the Benjamini-Hochberg method to control the False Discovery Rate (FDR). Genes were identified as DEGs based on a stringent q-value threshold of <0.05. The biological relevance of the identified DEGs was explored through Gene Ontology (GO) ORA via the clusterProfiler R package [76], applying a significance cutoff of q-value < 0.05. To further investigate the systemic interactions between protein products of the DEGs, we utilized the STRING database (Search Tool for the Retrieval of Interacting Genes/Proteins) v. 12.0 [77] accessed on 1 March 2026 to predict protein-protein interaction (PPI) networks. The resulting interactome was imported into Cytoscape v. 3.10.3 [78] for advanced visualization and topological analysis, allowing for the identification of highly connected hub genes and functional modules within the network.

### 4.6. Protein Isolation and Western Blotting Procedures

Western blot analyses were performed to evaluate selected autophagy-associated and oxidative-stress-related proteins under the same experimental conditions used for transcriptomic profiling. Differentiated SH-SY5Y cells were exposed to vehicle (DMSO) or 5 µM PCB 118 for 24 h. Protein extracts were obtained from independent biological replicates for each condition and processed separately for electrophoresis, transfer, immunodetection, and densitometric analysis. SH-SY5Y cells were collected after treatment by detaching them with a trypsin–EDTA solution. Nuclear and cytoplasmic proteins were then isolated using the NE-PER extraction kit (#78833, Thermo Scientific™, Waltham, MA, USA), following the manufacturer’s instructions, and total protein content was quantified by the Bradford colorimetric assay (Bio-Rad, Hercules, CA, USA).

For immunoblotting, equal protein amounts (30 µg per sample) were separated by SDS–polyacrylamide gel electrophoresis and transferred onto PVDF membranes (Immobilon-P, Merck Millipore, Darmstadt, Germany). Membranes were blocked for 1 h at room temperature in TBS containing 5% skim milk and subsequently incubated overnight at 4 °C with the selected primary antibodies: anti-p62 (#5114, 1:1000; Cell Signaling Technology, Danvers, MA, USA); anti-LC3 (#4108, 1:1000; Cell Signaling Technology, Danvers, MA, USA); anti-Beclin1 (#3738, 1:1000; Cell Signaling Technology, Danvers, MA, USA); anti-Sod1 (#HPA001401, 1:1250; Sigma-Aldrich, St. Louis, MO, USA); anti-PINK1 (#P0076, 1:1500; Sigma-Aldrich, St. Louis, MO, USA).

After washing, membranes were exposed for 1 h at room temperature to an HRP-conjugated anti-rabbit secondary antibody (1:2000; #sc-2357, Santa Cruz Biotechnology, Inc., Dallas, TX, USA). Protein bands were detected using Immobilon Forte Western HRP Substrate (Millipore, Burlington, MA, USA). Images were acquired with the ChemiDoc XRS+ System (Bio-Rad, Hercules, CA, USA), and densitometric analysis was performed using ImageJ version 1.54j. Membranes were subsequently stripped using Restore Western Blot Stripping Buffer (#21059, Thermo Scientific, Meridian, Rockford, IL, USA) and reprobed with an HRP-conjugated GAPDH antibody (#3683, 1:1000; Cell Signaling Technology, Danvers, MA, USA), which served as the loading CTRL.

### 4.7. Statistical Analysis

Statistical analyses were conducted using GraphPad Prism version 10.1 (GraphPad Software, La Jolla, CA, USA). Group differences were assessed using one-way ANOVA, followed by Bonferroni’s post hoc test for multiple comparisons. A *p*-value ≤ 0.05 was considered statistically significant. Data are expressed as the mean ± standard deviation.

## 5. Conclusions

In conclusion, the present study demonstrates that PCB 118 exposure, even under non-cytotoxic conditions, is sufficient to induce a marked transcriptional reprogramming in human SH-SY5Y neuron-like cells. RNA-seq profiling revealed that PCB 118 primarily targets chromatin-related pathways, including nucleosome organization, chromatin remodeling, and epigenetic regulatory processes, while concomitantly repressing gene networks involved in ribosome biogenesis, ncRNA processing, and ribonucleoprotein complex assembly. Overall, these findings point to an early disruption of transcriptional and translational homeostasis, consistent with a neuronal stress-associated molecular phenotype that may increase vulnerability to dysfunction.

A major strength of this study lies in the transcriptomic strategy employed, which enabled the simultaneous investigation of a broad range of genes and biological pathways, thereby providing an integrated overview of the molecular programs altered by PCB 118. The use of stringent filtering criteria further enhanced the robustness of the analysis by minimizing false-positive associations and facilitating the identification of the most robustly modulated pathways. Within this framework, the protein-level findings should be interpreted as supportive biological validation of selected downstream processes rather than as the primary evidence of toxicity. In addition, although changes in p62, total LC3, and Beclin-1 suggest an alteration of autophagy-associated markers, autophagic flux was not directly assessed. Therefore, these findings should be interpreted cautiously and cannot distinguish between increased autophagosome formation, impaired autophagosome degradation, or a mixed adaptive response. At the same time, the exploratory nature of the study represents an important limitation, as the mechanisms inferred from transcriptomic profiling still require functional validation. However, the RNA-seq analysis revealed a clear separation between control and PCB 118-treated samples and identified coherent pathway-level signatures. Therefore, the transcriptomic findings should be interpreted as hypothesis-generating and require validation in larger independent datasets and complementary experimental models. Further investigations in primary neuronal cultures or in vivo models will be necessary to better define the physiological and translational relevance of these alterations.

**Figure 9:**
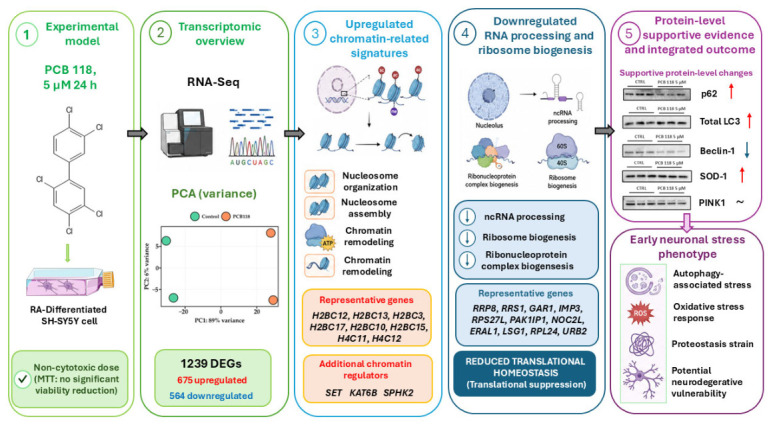
Integrated overview of PCB 118-induced molecular changes in differentiated SH-SY5Y cells. RA-differentiated SH-SY5Y cells were exposed to a non-cytotoxic concentration of PCB 118, followed by RNA-seq analysis and protein-level assessment. Transcriptomic profiling identified 1239 DEGs and revealed two main signatures: upregulation of chromatin-related pathways and downregulation of ncRNA processing, ribonucleoprotein complex biogenesis, and ribosome biogenesis. Selected Western blot markers further supported the presence of autophagy-associated and oxidative-stress-related responses, contributing to an early neuronal stress phenotype. This image was created using the image bank of Servier Medical Art (available online: http://smart.servier.com/, accessed on 14 May 2026), licensed under a Creative Commons Attribution 4.0 (CC BY 4.0), available online: https://creativecommons.org/licenses/by/4.0/ (accessed on 14 May 2026). Chemical structures depicted in the figure were generated using the PubChem database (accessed on 14 May 2026). PCB: Polychlorinated biphenyl; RA: Retinoic acid; MTT: 3-(4,5-dimethylthiazol-2-yl)-2,5-diphenyltetrazolium bromide assay; RNA-seq: RNA sequencing; PCA: Principal component analysis; CTRL: Control; DEGs: Differentially expressed genes.

Altogether, our data identify transcriptomic dysregulation as the core early response to PCB 118 exposure in neuron-like cells, with downstream alterations in proteostasis-related pathways supporting the biological relevance of this molecular perturbation (Figure 9). Future studies should address the temporal dynamics of PCB 118-induced molecular responses and explore the use of selective pathway inhibitors to establish causal links between the dysregulated signaling and epigenetic mechanisms identified in the present study.

## Figures and Tables

**Table 1:** Significantly enriched GO pathways across downregulated DEGs.

Ontology	Description	q-Value	N. of DEGs
BP	ncRNA processing	8.3 × 10^−3^	37
BP	Ribonucleoprotein complex biogenesis	8.3 × 10^−3^	39
BP	Ribosome biogenesis	3.9 × 10^−2^	28

**Table 2:** Significantly enriched GO pathways using all DEGs, all nested in GO obtained from Over-ORA using just upregulated DEGs.

Ontology	Description	q-Value	N. of DEGs
BP	Nucleosome organization	1.8 × 10^−2^	27
BP	Nucleosome assembly	1.8 × 10^−2^	22
BP	Chromatin remodeling	3.1 × 10^−2^	50
CC	Nucleosome	9.1 × 10^−3^	23
CC	Protein–DNA complex	1.7 × 10^−2^	32
MF	Structural constituent of chromatin	1.1 × 10^−2^	19

## Data Availability

The data presented in this study are openly available in the NCBI Sequence Read Archive at BioProject; accession number: PRJNA1435793, PRJNA1378135.

## Supplementary Materials

The following supporting information can be downloaded at: https://www.mdpi.com/article/10.3390/ijms27115058/s1.

## Abbreviations

The following abbreviations are used in this manuscript in the reported order:

PCBs	Polychlorinated biphenyls
POPs	persistent organic pollutants
CNS	central nervous system
NDL-PCBs	non-dioxin-like PCBs
DL-PCBs	dioxin-like PCBs
AhR	aryl hydrocarbon receptor
TCDD	2,3,7,8-tetrachlorodibenzo-p-dioxin
AD	Alzheimer Disease
RNA-seq	RNA sequencing
DMSO	Dimethyl sulfoxide
RA	retinoic acid
DEGs	differentially expressed genes
GLM	generalized linear models
FDR	False Discovery Rate
GO	Gene Ontology
ORA	Over-Representation Analysis
STRING	Search Tool for the Retrieval of Interacting Genes/Proteins
PPI	protein-protein interaction
CTRL	untreated control
PCA	Principal Component Analysis
FC	Fold Change
BP	Biological processes
MF	molecular function
CC	cellular component
UPS	ubiquitin-proteasome system
ALP	autophagy-lysosome pathway
sHSPs	small heat-shock proteins
ATG	autophagy-related proteins
MTT	3-(4,5-dimethylthiazol-2-yl)-2,5-diphenyltetrazolium bromide assay
