# Supplementary figures and images for: PCB 118 Exposure Modulates Chromatin Organization, Ribosome Biogenesis, and Autophagy-Related Pathways in Neuron-like: A Transcriptomic Analysis
Source: Int J Mol Sci. 2026 Jun 3;27(11):5058. doi: 10.3390/ijms27115058 (PMC13256503; doi:10.3390/ijms27115058)

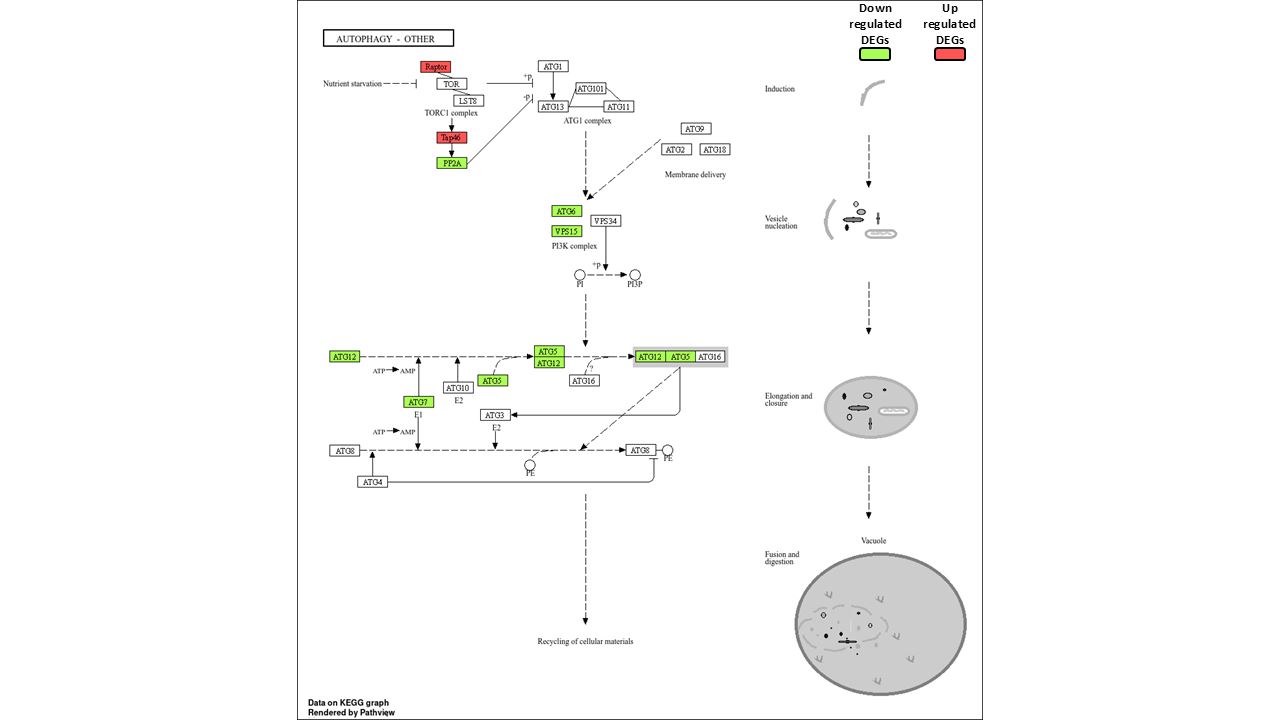

Supplement: Supplementary file 1 [file ijms-27-05058-s001.zip › Supplementary Figure S1.jpg]

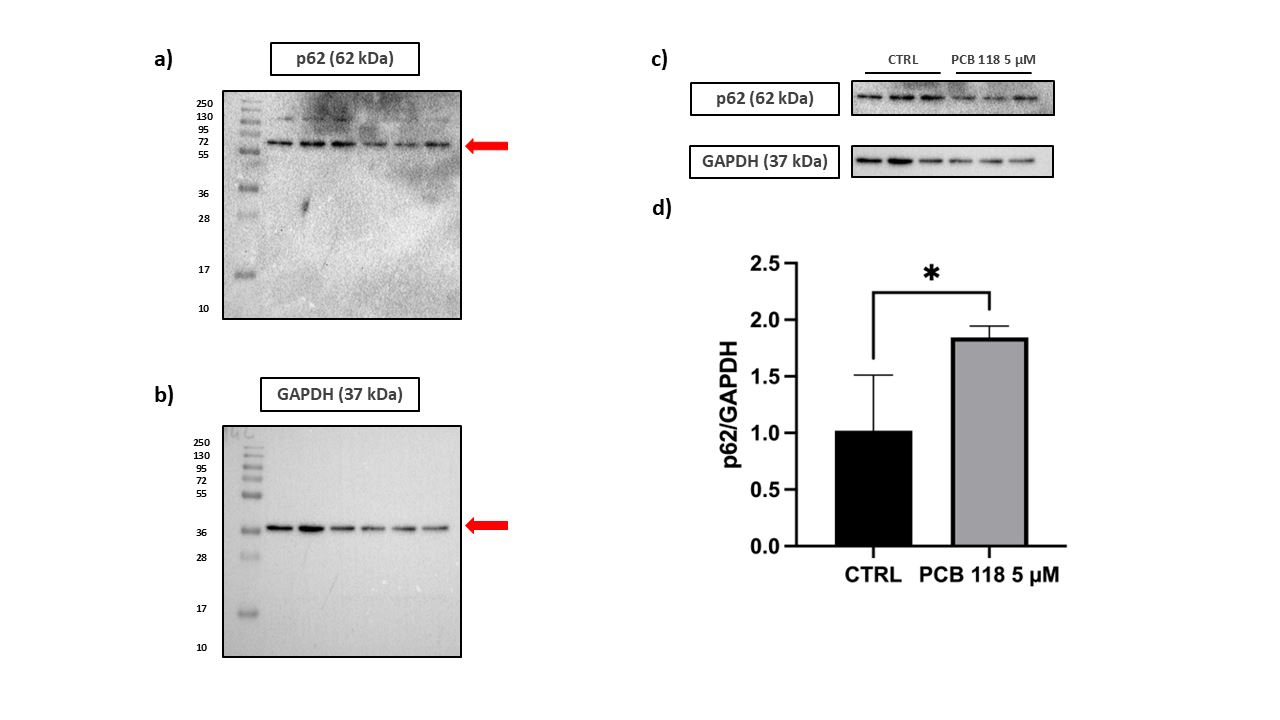

Supplement: Supplementary file 1 [file ijms-27-05058-s001.zip › Supplementary Figure S2.jpg]

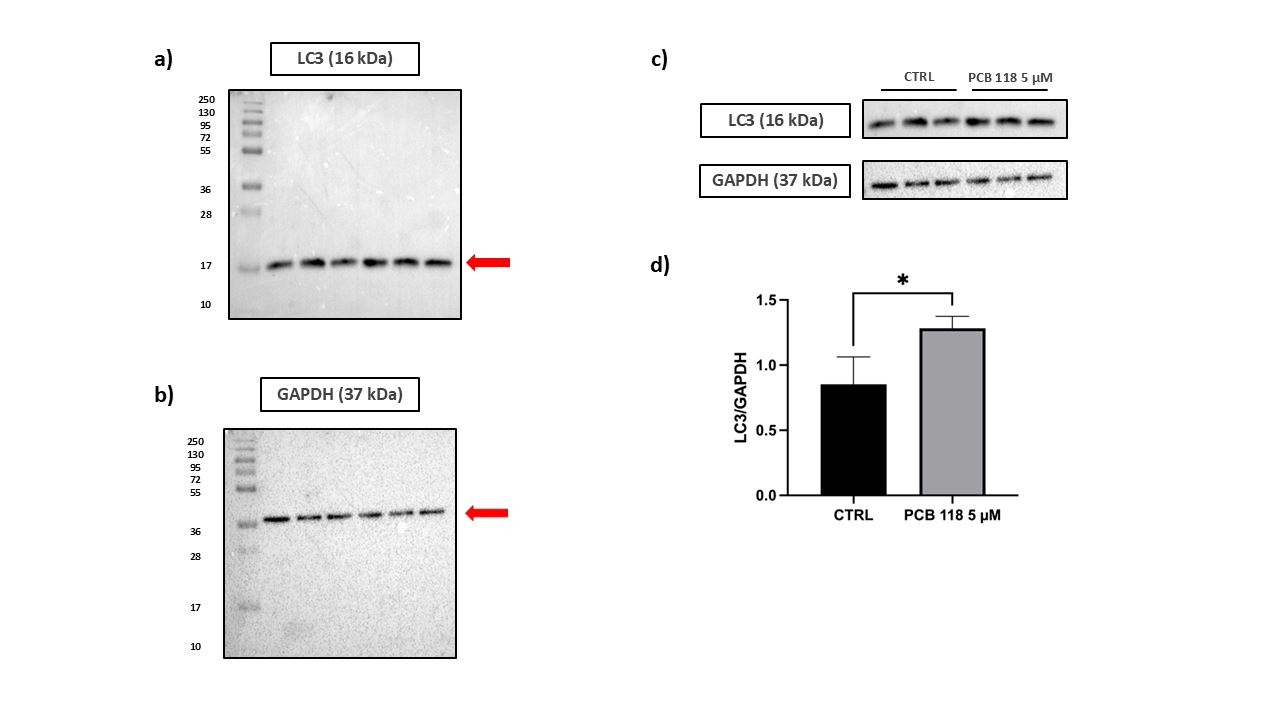

Supplement: Supplementary file 1 [file ijms-27-05058-s001.zip › Supplementary Figure S3.jpg]

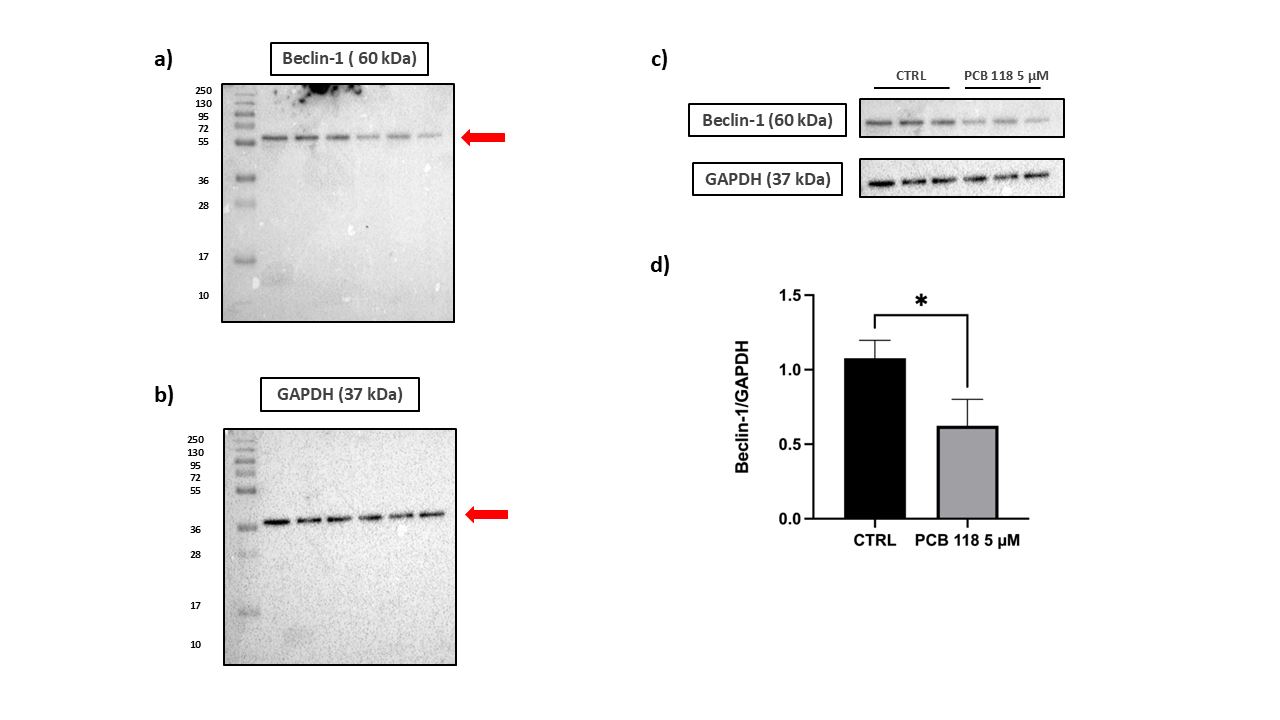

Supplement: Supplementary file 1 [file ijms-27-05058-s001.zip › Supplementary Figure S4.jpg]

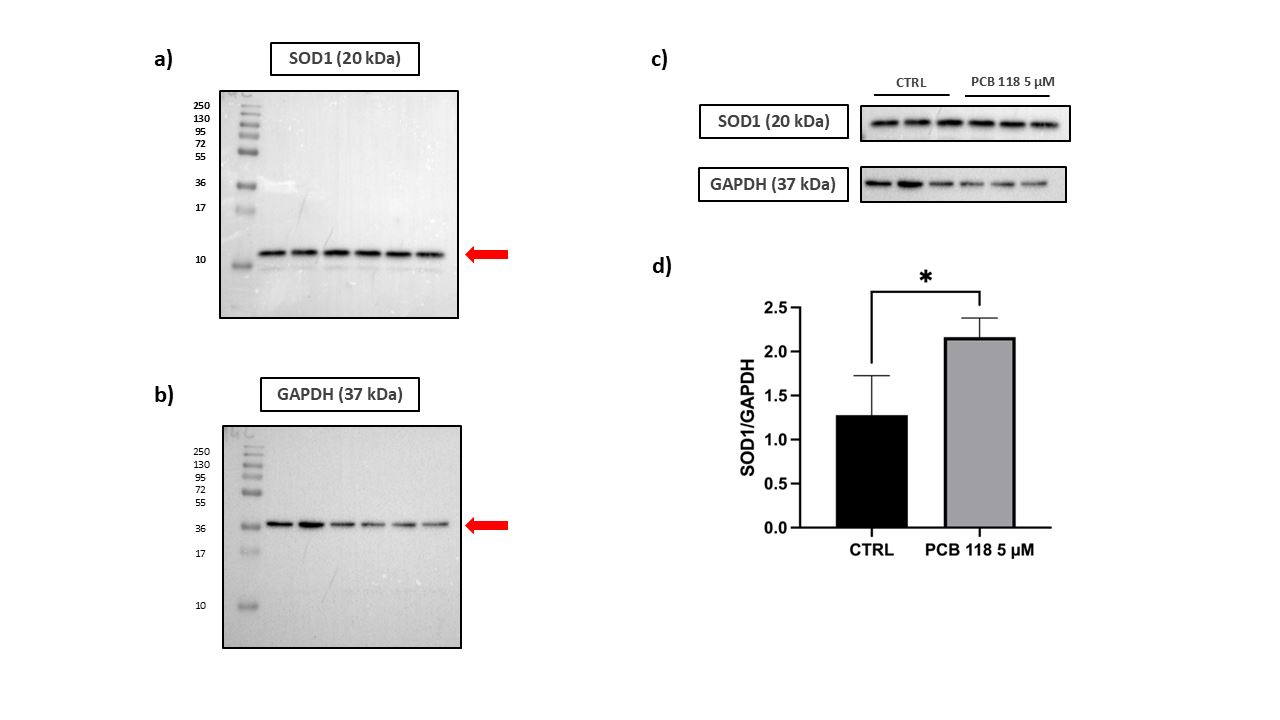

Supplement: Supplementary file 1 [file ijms-27-05058-s001.zip › Supplementary Figure S5.jpg]

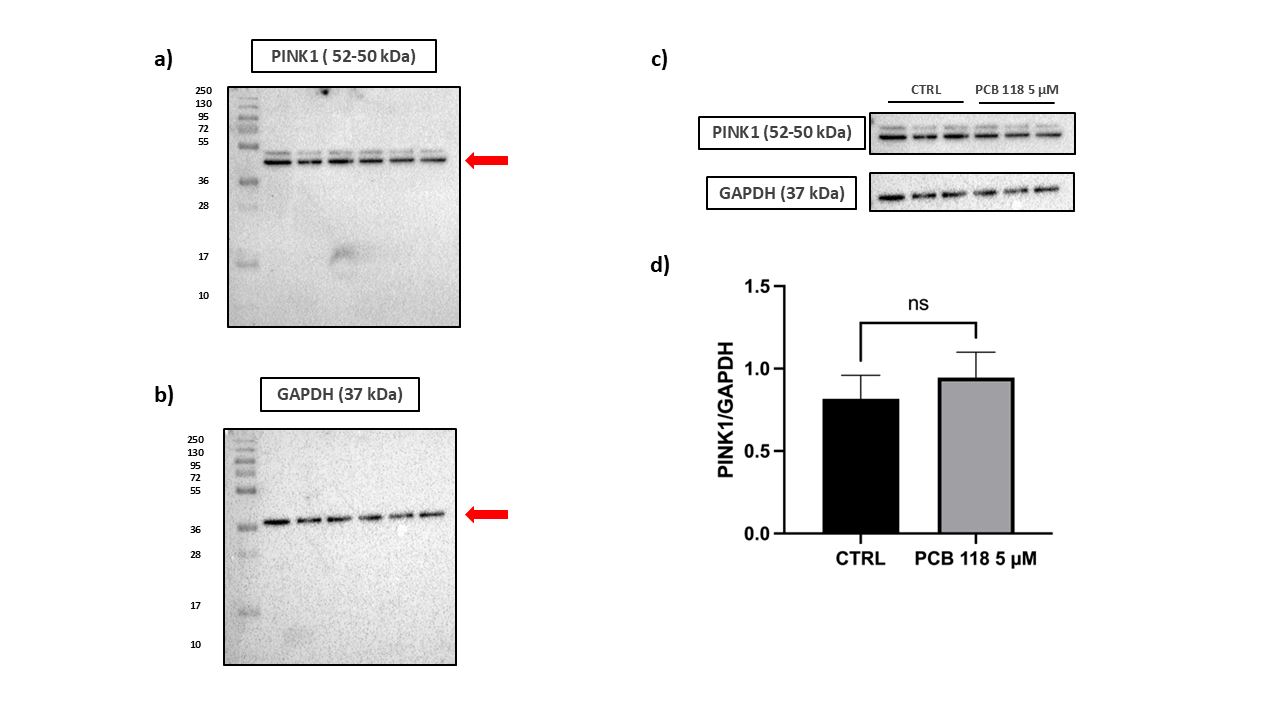

Supplement: Supplementary file 1 [file ijms-27-05058-s001.zip › Supplementary Figure S6.jpg]
